# Somatic Malignant Transformation of a Testicular Teratoma: A Case Report and an Unusual Presentation

**DOI:** 10.1155/2019/5273607

**Published:** 2019-11-03

**Authors:** Dalia Y. Ibrahim, Hongliu Sun

**Affiliations:** Department of Pathology, University of Toledo Medical Center, Toledo, OH, USA

## Abstract

Testicular cancer represents 1% of all malignant tumors in men. About 95% of testicular cancers are germ cell tumors (GCTs). These can be divided into nonseminomatous GCTs (NSGCTs) and seminomas. NSGCTs include teratomas, yolk sac tumors, embryonal carcinomas, choriocarcinomas, and mixed tumors. Only 2–6% of testicular teratomas are pure teratomas. Pure teratomas can be subdivided into prepubertal and postpubertal. The prognosis is significantly different between these two age groups. Different from teratomas in ovary, the immaturity in a teratoma is not an indication of their biologic behavior; the age of the patient is of greater importance. Malignant transformation of teratoma occurs in only 3–6% of testicular GCTs. The most frequent transformed histologic types consist of rhabdomyosarcoma, adenocarcinoma, and primitive neuroectodermal tumors. We report a rare case of pure postpubertal testicular teratoma with a secondary somatic malignancy that was an incidental finding in a patient presenting with lower back pain and testicular torsion.

## 1. Background

Primary testicular tumors are rare, but they are the most common solid malignancy in men 20–35 years of age [1]. Over the past decade, their incidence has increased approximately 1.2% per year. Testicular germ cell tumors (TGCTs) account for about 95% of all testicular tumors. Their diagnosis depends on physical examination, imaging, serum tumor markers and pathological examination. Standard treatment is radical orchiectomy and/or combination of chemotherapy, radiotherapy or retroperitoneal lymph node dissection [2].

Amplification of chromosome arm 12p, such as isochromosome 12p, is nearly universal in TGCTs. The pathogenesis of TGCTs is complex. It is believed that they arise from premalignant intratubular germ cell neoplasia (IGCN), which in turn develops from failure of normal maturation of fetal gonocytes [2].

Testicular teratomas have been divided into 2 groups: prepubertal and postpubertal [3]. The prepubertal type is a benign childhood tumor, does not recur, and does not metastasize. Postpubertal teratomas are defined by the WHO as typically part of mixed germ cell tumors and rarely pure neoplasms, almost always malignant and usually present at a metastatic site [4, 5].

Malignant transformation of teratoma is rare, occurring in only 3–6% of TGCTs. The most frequent transformed histologic types consist of rhabdomyosarcoma, adenocarcinoma, and primitive neuroectodermal tumors [6]. They are more aggressive than teratomas without malignant transformation, usually metastatic at presentation, and have a high recurrence rate. The main therapy for localized disease is surgical resection because they are resistant to radiation and systemic chemotherapy.

We report a rare case of pure postpubertal testicular teratoma with a secondary somatic malignancy that was an incidental finding in a patient presenting with lower back pain and testicular torsion.

## 2. Case Report

Patient is a 38-year-old white male with a past medical history significant only for schizophrenia, currently on Abilify. He presented to the emergency department at the University of Toledo Medical Center with lower abdominal and back pain (Pain scale = 4/10) that had been intermittent for the past 2 weeks, with no particular inciting factor. On further questioning, he admits that a week earlier he fell and experienced sharp pain in his testicle. This pain was severe but subsequently subsided. Physical examination revealed height of 69 inches and weight of 220 pounds. His vital signs were within normal limits. The remaining physical examination was normal except for a firm enlarged left scrotum, which is nontender. Patient was given pain medications and imaging was ordered.

Bedside duplex ultrasound showed unremarkable right testis with good Doppler flow. However, the left testis had minimal to no blood flow and was extremely heterogeneous with small fluid collection, which was concerning for testicular torsion.

**Figure 1:**
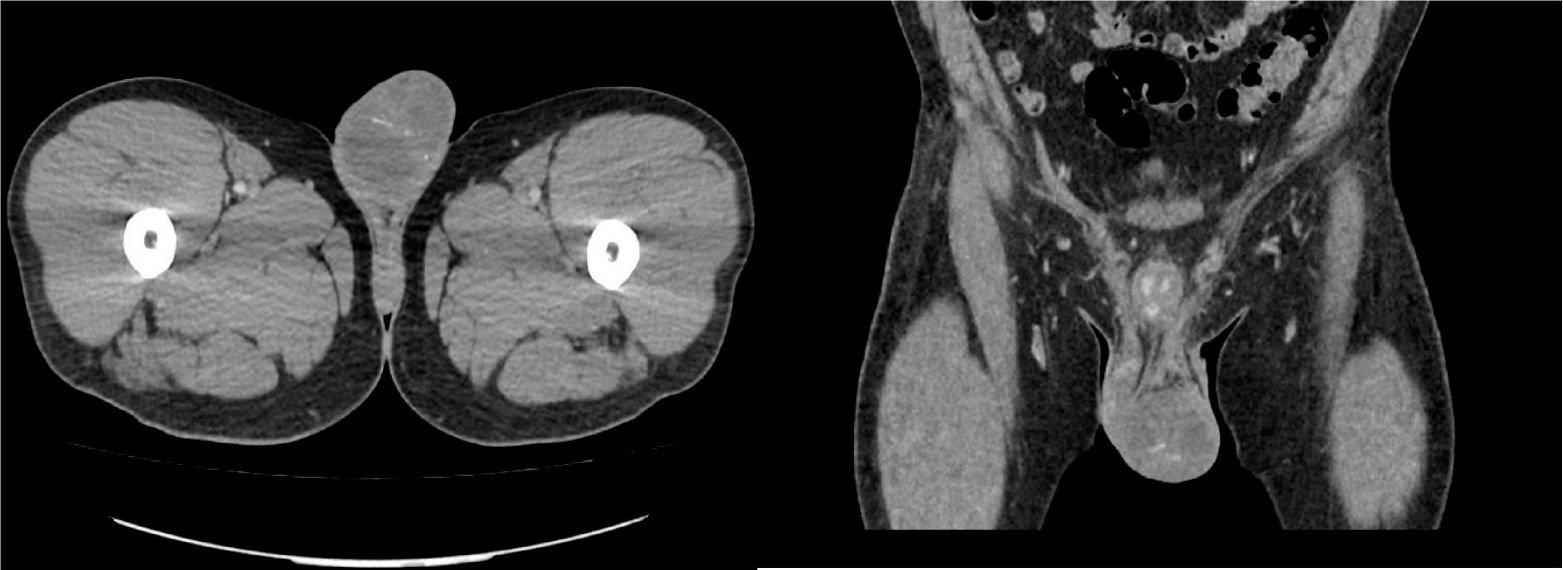
CT of the abdomen and pelvis: enlarged contents of the left hemiscrotum with calcifications (arrow).

Testicular mass could not be excluded from the ultrasound study. Hence serum beta HCG, ALP, AFP were ordered, all of which were within normal range. In addition, CT of the chest, abdomen and pelvis with contrast showed left adrenal mass measuring a maximum of 1.9 cm, most likely a simple adenoma. There was no evidence of retroperitoneal or inguinal adenopathy. The left hemiscrotum showed enlarged contents with calcifications (Figure 1). A diagnosis of testicular torsion was made and urgent scrotal exploration and hemiorchiectomy was performed. Postoperative course was uneventful and patient was discharged on Percocet, Colace, and Keflex. He was also advised to follow up with his doctor for the pathology results.

**Figure 2:**
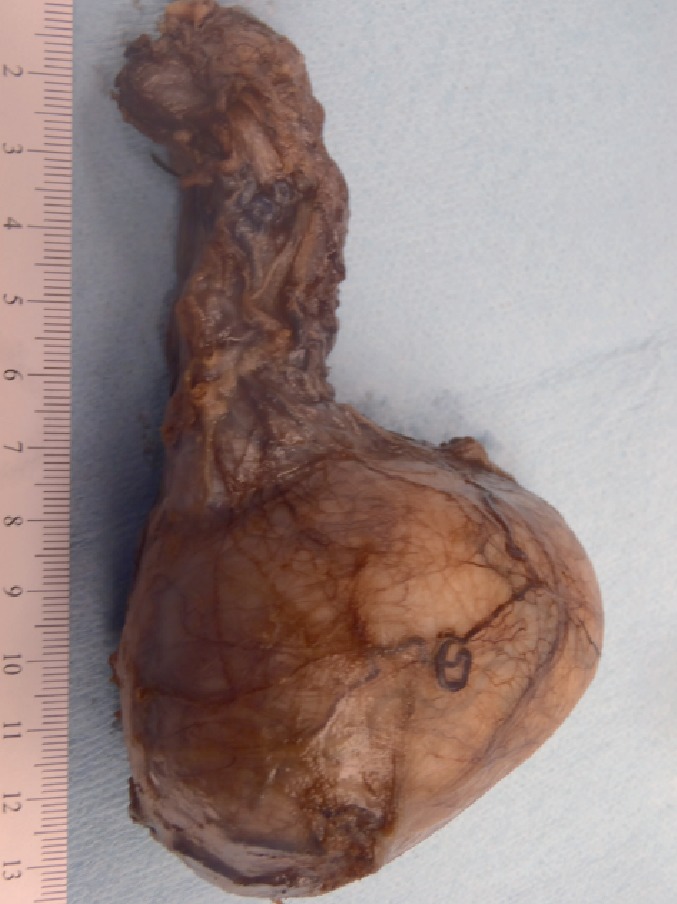
Gross picture showing enlarged left testis.

**Figure 3:**
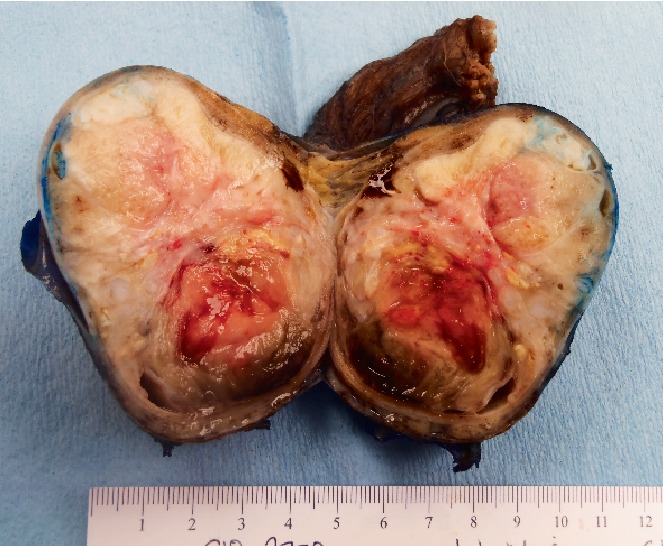
Cut surface of the testicular mass showing areas of necrosis, calcification, and cystic area.

On gross examination, the testicle was enlarged (Figure 2), measuring 7 × 5.4 × 5 cm, and the spermatic cord was 6.7 × 2.6 cm. Tunica vaginalis and albuginea showed no apparent gross invasion. The spermatic cord showed no gross abnormalities. Cut surface (Figure 3) showed a well-circumscribed variegated mass, measuring 7.0 × 5.2 × 4.8 cm. There were focal areas of calcifications and necrosis. In addition, there was a cystic structure (1.2 × 0.8 × 0.8 cm) filled with tan serous fluid.

**Figure 4:**
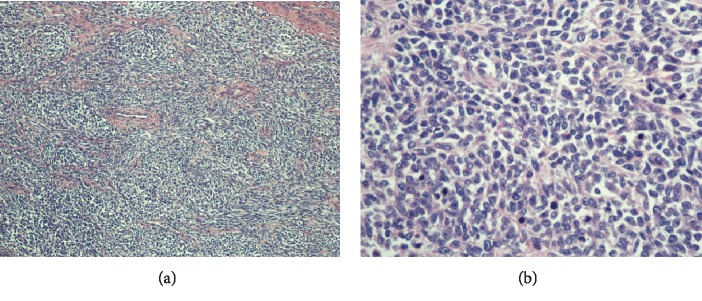
Low power (a) and high power (b) views showing malignant spindle cells with abundant mitoses.

**Figure 5:**
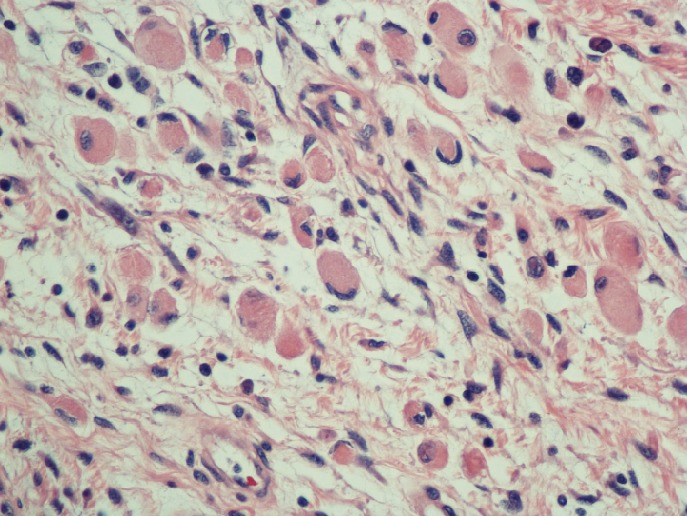
Areas of tumor showing rhabdomyoblastic differentiation.

**Figure 6:**
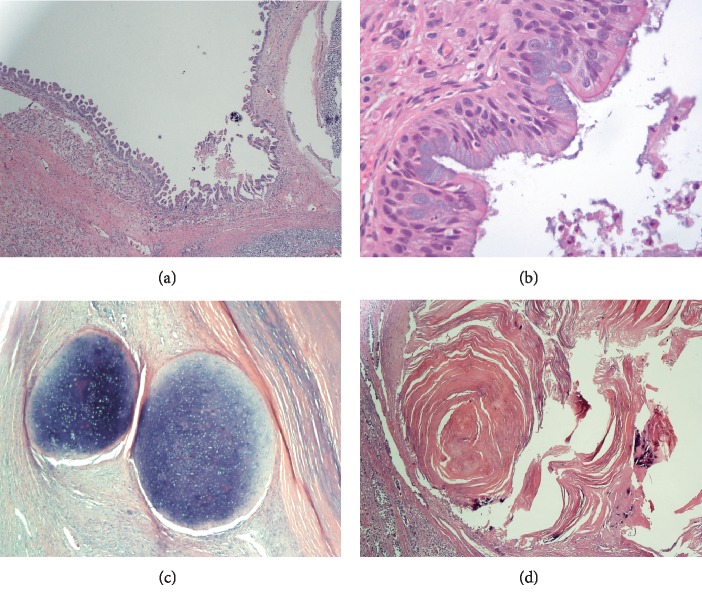
Teratomatous components: glandular epithelium (a), ciliated columnar epithelium (b), cartilage (c), and epidermoid cyst (d).

**Figure 7:**
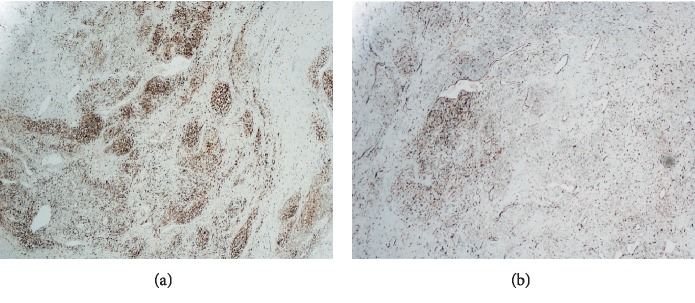
Tumor showing positive desmin (a) and vimentin (b).

**Figure 8:**
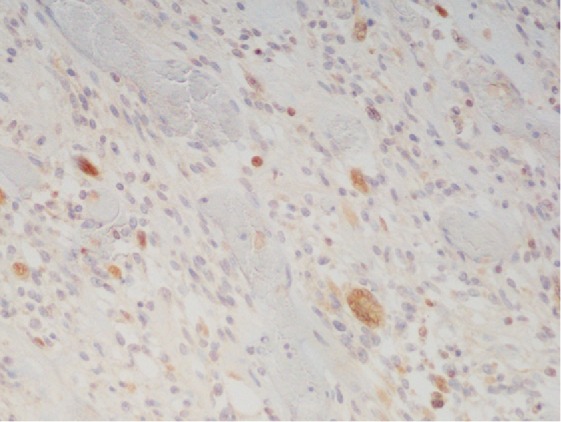
Myo-D positive in rhabdoid cells.

Microscopic examination of the mass revealed predominantly overt malignant spindled cells displaying pleomorphism, hyperchromasia, and increased mitosis, with extensive necrosis in the background (Figure 4). There were cells with abundant eosinophilic cytoplasm, some of which with striations, indicating rhabdomyoblastic differentiation (Figure 5). Scrutinous searching further identified different benign tissues including cartilage, squamous epithelium, and rare bone, respiratory and glandular epithelium (Figure 6). No other components of GCT or intratubular germ cell neoplasia were seen. The tumor was limited to the testis, and spermatic cord was not involved. However, extensive lymphovascular invasion was present. Immunohistochemistry revealed that the tumor cells were positive for desmin and vimentin (Figure 7). The rhabdoid cells were positive for myogenin and focally weakly positive for MyoD (Figure 8). A final diagnosis of teratoma with somatic-type malignancy: rhabdomyosarcoma was made.

## 3. Discussion

Malignant transformation of teratoma is defined as the transformation of a somatic teratomatous component of a germ cell tumor (GCT) to a nongerm cell malignant tumor [7]. It was first reported by Waldeyer in as early as 1868. However, it was not until 1977 that WHO classified a germ cell neoplasm with nongerm cell elements as a teratoma with malignant transformation [8].

The most common examples of malignant transformation are sarcoma (e.g., rhabdomyosarcoma, osteosarcoma, chondrosarcoma, angiosarcoma, and liposarcoma), carcinoma (adenocarcinoma and squamous cell carcinoma), primitive neuroectodermal tumor, as well as hematologic malignancies [9].

A pitfall in histopathological diagnosis is the potential of misinterpreting atypical epithelial or stromal cells for malignant transformation. Davey et al. established that cytological atypia with no expansile growth and infiltration of the surrounding tissue is not enough to make the diagnosis. Carcinoma or sarcoma component should consist of an expansile component that is exclusively occupying at least one low-power field (4× objective, 5 mm in diameter) [10, 11].

Unlike conventional GCTs, which respond significantly to platinum-based chemotherapy with an estimated 90% survival, teratoma with malignant transformation is a very aggressive tumor that is resistant to chemotherapy and is usually metastatic at presentation [9, 12]. It was concluded from several studies that there is no association between prognosis and tumor type, the degree of differentiation, or the percentage of the specific subtypes in relation to the amount of other germ cell components. The clinical stage is the most important prognostic factor. Patients with stage I (disease confined to the testis) had an excellent outcome. Combined stages I and II disease had significantly better outcome than stage III. Surgical resection combined with chemotherapy appears to be the therapy of choice [7].

This case report signifies the importance of maintaining a broad differential with a testicular torsion presentation and includes proper histopathologic diagnosis and impact of early diagnosis on prognosis of testicular teratoma with malignant transformation.
